# Estrogen and Progesterone Receptor Expression in Breast Carcinoma and Its Association With Clinicopathological Variables Among the Pakistani Population

**DOI:** 10.7759/cureus.9751

**Published:** 2020-08-14

**Authors:** Shahzada K Sohail, Rahat Sarfraz, Muhammad Imran, Muhammad Kamran, Samina Qamar

**Affiliations:** 1 Basic Medical Sciences, College of Medicine, University of Bisha, Bisha, SAU; 2 Pathology, King Edward Medical University, Lahore, PAK; 3 Pathology, Allama Iqbal Medical College, Lahore, PAK; 4 Pathology, Federal Government Poly Clinic Postgraduate Medical Institute, Islamabad, PAK

**Keywords:** carcinoma breast, estrogen receptor, progesterone receptor

## Abstract

Introduction

The prognosis of breast cancer depends on the histological type, size of the tumor, tumor necrosis, skin, nipple and chest wall invasion, lymphovascular invasion, grade, stage, the status of estrogen receptor (ER), progesterone receptor (PgR), and human epidermal growth factor receptor 2 (HER2), cell proliferation marker (ki-67), and type of therapy. Estrogen receptor and progesterone receptor expression in breast cancer is, so far, the most useful predictive marker. We have undertaken this study to find the expression of ER and PgR in breast carcinoma and its association with other prognostically important clinicopathological variables.

Materials and methods

In this cross-sectional study, a total of 130 cases of modified radical mastectomy that have been diagnosed as malignant on histopathology were collected from the pathology department of Allama Iqbal Medical College, Lahore, from January 2016 to May 2018. The demographic data and gross and microscopic findings were recorded. Immunohistochemistry (ER, PgR) was applied to suitable tumor sections and their status was evaluated semi-quantitatively by histopathologists using College American Pathologist (CAP) guidelines.

Result

Most of the breast cancer patients (69; 53.1%) were below 50 years of age. Fifty-nine (45.4%) and 48 (36.9%) cases were positive for ER and PgR, respectively, showing lower hormonal receptor positivity than that reported in the western population where ER expression has been found in 50%-80% of cases and PR expression is found in 60%-70% of cases of invasive ductal carcinoma. The association of the expression of hormone receptors with a clinicopathological variable was demonstrated. ER-/PgR- tumors showed a higher histologic grade, greater tumor size, and more lymph node involvement by metastasis.

Conclusion

Low hormone receptor positivity is associated with young patients, advanced stage at presentation, and higher grade in our population. The tumor characteristics are different as compared to the western population. This suggests more consideration to the screening, early diagnosis, and molecular or immunohistochemical typing of this cancer in our population.

## Introduction

Breast carcinoma is the most common organ malignancy in females worldwide and has also been ranked second in cancer mortality among female patients [1-2]. Breast cancer accounts for 24.4% of all cancers in the female Pakistani population while in the west, it accounts for 23% of cases [3-4]. Studies show that one out of nine Pakistani females is suffering from breast cancer [3-4]. Breast carcinoma is a heterogeneous disease with diverse histopathological types and molecular and clinical features. The prognosis and treatment response of the patient is dependent on many factors. Prognostically, the most important factors are the histological type, size of the tumor, tumor necrosis, skin, nipple and chest wall invasion, lymphovascular invasion, grade, stage, estrogen receptor (ER) status, progesterone receptor (PgR), human epidermal growth factor receptor 2 (HER2), cell proliferation marker (ki-67), and type of therapy [4,5-6].

Estrogen receptor and progesterone receptor expression in breast cancer is so far the most useful predictive marker [1,2,5]. ER and PgR are intercellular steroid hormone receptors and have been frequently demonstrated in breast carcinoma. ER expression has been found in 50%-80% of cases and PgR expression is found in 60%-70% of cases of invasive ductal carcinoma [7-8]. Tumors expressing ER and PgR receptors show a good response to hormonal therapy and chemotherapy leading to a better prognosis, good survival, and less mortality [1,4,9-10]. Studies from Pakistan and India show a higher rate of hormone receptor-negative tumors than that found in the western population [2-3,6].

Other than hormonal receptors, the most promising biomarker in breast cancer is HER2. This is a protein that is overexpressed in 18%-20% of breast cancers and has prognostic as well as predictive value [2,7-8]. Tumors expressing HER2 are histologically high grade, are most likely to metastasize, and have a worse prognosis [10]. However, HER2 overexpression also predicts a good response to anti-HER2 agents, including trastuzumab and lapatinib [4-5,7].

Breast carcinoma with the same histologic type, grade, and stage may show different outcomes and different responses to therapy. Evaluation of prognostic and predictive biomarkers ER, PgR, and HER2 is recommended in every case of breast carcinoma [5-7]. Immunohistochemistry (IHC) is the most widely employed method to determine the status of ER, PgR, and HER2 in formalin-fixed paraffin-embedded tissue samples of breast cancers [7].

Our main challenge in every case of breast carcinoma is the non-affordability of the patients, fewer resources, and the non-availability of immunohistochemistry in public sector institutes.

The present study aims to find the expression of ER and PgR in breast carcinoma and its association with prognostically important clinicopathological variables. This information will help in understanding the biological and clinical behavior of breast cancer in our population.

## Materials and methods

In this cross-sectional study, a total of 130 cases of modified radical mastectomy that have been diagnosed as malignant on histopathology were collected from the pathology department of Allama Iqbal Medical College, Lahore, from January 2016 to May 2018.

The demographic data and gross and microscopic findings were recorded following standard protocols for handling and reporting the specimens. Immunohistochemistry (ER, PgR) was applied to suitable tumor sections and their status was evaluated semi-quantitatively by histopathologists using College American Pathologist (CAP) guidelines. On immunohistochemistry, ER and PgR were interpreted as positive when the tumor cells showed positive nuclear staining in at least 1% of the tumor cells.

The data of the patients with incomplete histopathological, surgical details, and with no/incomplete information about ER and PgR status were excluded from the study.

Data of all the patients were compiled and assessed for the pattern of expression of ER/PgR in invasive ductal carcinoma breast.

The association of ER and PgR status with clinicopathological variables, age, tumor grade, tumor size, necrosis, lymphovascular invasion, and lymph node status, was also assessed. P-value was calculated using the chi-square test, taking a significance level of <.05.

## Results

A total of 130 cases of modified radical mastectomy were included in this study. The ages of the patients ranged from 24 to 78 years. Most of the patients (53.1%) were below 50 years of age. Females comprised 98.4% of the cases. The male to female ratio was 1:64. The mean age of the respondents was 47.2±10.5 SD (Table 1).

**Table 1:** Age distribution among breast cancer patients

Age in Years	Frequency n=130	Percentage (%)
20-29	5	3.9%
30-39	18	13.8%
40-49	46	35.4%
50-59	44	33.8%
>60	17	13.1%
Total	130	100%
Mean	47.2±10.5SD	

**Figure 1:**
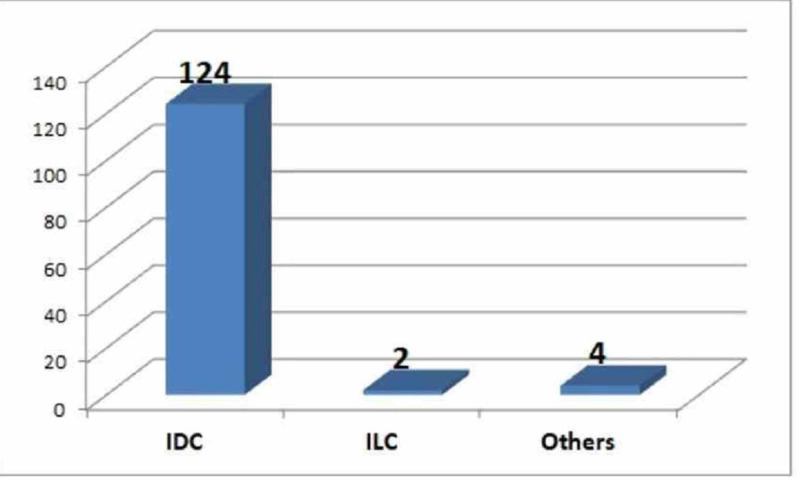
Frequency of different types of breast carcinoma

Invasive ductal carcinoma, not otherwise specified, was diagnosed in 124 (95.4%) of the cases and thus constituted the most predominant histological type. Two cases (1.5%) had invasive lobular carcinoma. One case each of medullary carcinoma, invasive ductal carcinoma with mucinous features, adenoid cystic carcinoma, and a solid variant of papillary carcinoma were identified (Figure 1).

**Figure 2:**
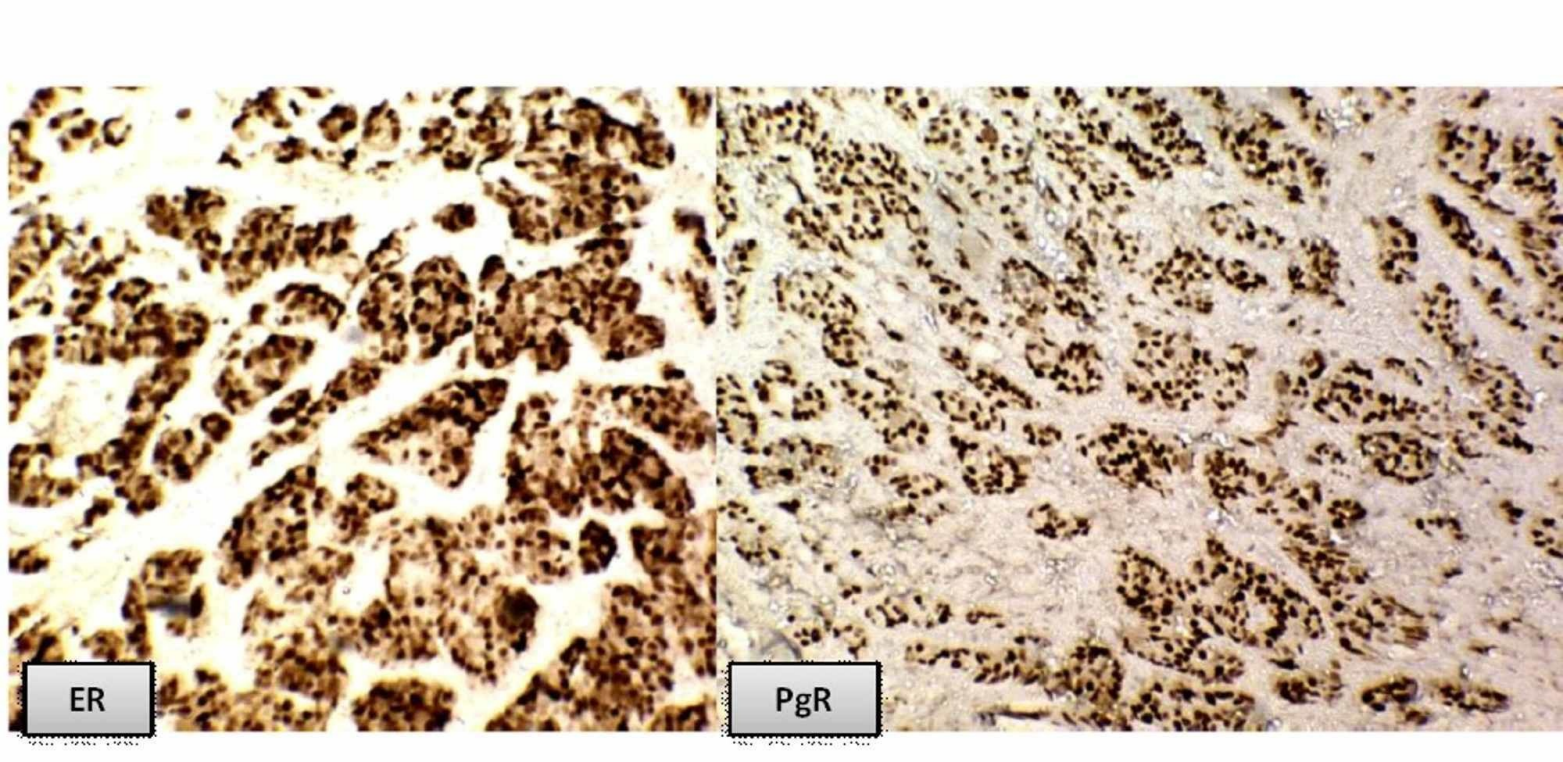
Breast invasive ductal carcinoma showing ER and PgR positivity

Fifty-nine (45.4%) cases showed positivity for ER. Forty-eight (36.9%) cases showed positivity for PgR. All the tumors were graded according to the modified Scarff-Bloom-Richardson grading system. Histologic Grade 3 predominated with 66 (50.8%) cases. Grades 1 and 2 were found in 14 (10.8%) and 50 (38.4%) cases, respectively. Forty-five (34.6%) and 50 (38.5%) cases of histologic Grade 3 are ER- and PgR-. Close to two-thirds of the cases (74; 56.9%) of tumors had size >5 cm. Forty-four (33.8%) cases had a size ranging from 2 cm to 5 cm and only 12 cases (9.2%) had a size less than 2 cm. Out of 74 cases with a greater than 5 cm size, 51 (39.2%) were negative for ER and 54 (41.5%) were negative for PgR receptors. Lymphovascular invasion and tumor necrosis were seen in 53 (40.8%) and 58 (44.6%) cases, respectively. The majority of these tumors are ER- and PgR-. Ninety cases (69.2%) showed metastatic tumor deposits in the axillary lymph nodes. One to three lymph nodes (Stage 1) were involved in 48 (36.9%) cases. The cases that involved four to nine lymph nodes (Stage 2) were 32 (24.6%) out of which 21 (16.2%) and 23 (17.7%) were ER and PgR negative, respectively. The cases that involved more than nine lymph nodes (Stage 3) were 10 (7.7%) out of which 9 (6.9%) were negative for both ER and PgR receptors (Table 2, Figure 2).

**Table 2:** Association of hormone receptors with clinicopathological variables

Clinicopathological variables		ER+ (n=59) (45.4%)	ER- (n=71) (54.6%)	p-value	PgR+(n=48) (36.9%)	PgR-(n=82) (63.1%)	p-value
Age	< 50 years	24 (18.5%)	45(34.6%)	0.0098	20(15.4%)	49(37.7%)	0.046
	≥50 years	35 (26.9%)	26(20%)		28(21.5%)	33(25.4%)	
Grade	Grade I	10(7.7%)	4(3.1%)	0.004	8(6.1%)	6(4.6%)	0.008
	Grade II	28(21.5%)	22(16.9%)		24(18.5%)	26(20%)	
	Grade III	21(16.2%)	45(34.6%)		16(12.3%)	50(38.5%)	
Tumor size	<2cm	7(5.4%)	5(3.9%)	0.0007	4(3.1%)	8(6.1%)	0.01
	2-5cm	29(22.3%)	15(11.5%)		24(18.5%)	20(15.4%)	
	> 5cm	23(17.7%)	51(39.2%)		20(15.4%)	54(41.5%)	
Lymphovascular invasion	Present	16(12.3%)	37(28.5%)	0.004	9(6.9%)	44(33.9%)	0.0009
	Absent	43(33.1%)	34(26.1%)		39(30%)	38(29.2%)	
Necrosis	Present	19(14.6%)	39(30%)	0.009	15(11.5%)	43(33.1%)	0.019
	Absent	40(30.8%)	32(24.6%)		33(25.4%)	39(30%)	
Lymph node stage	0	18(13.8%)	22(16.9%)	0.01	15(11.5%)	25(19.2%)	0.055
	1	29(22.3%)	19(14.6%)		24(18.5%)	24(18.5%)	
	2	11(8.5%)	21(16.2%)		9(6.9%)	23(17.7%)	
	3	1(0.8%)	9(6.9%)		1(0.8%)	9(6.9%)	

According to the results of hormone receptors, all the cases were divided into the following four groups: (ER+, PgR+), (ER+, PgR-), (ER-, PgR+), and (ER-, PgR-). Positivity for both the receptors was seen in 40 (30.8%) cases while both receptors were negative in 63 (48.5%) cases. The ER+, PgR- and ER-, PgR+ groups had 19 (14.6%) and eight (6.1%) cases, respectively (Table 3).

**Table 3:** Proportion of hormone receptor types

Sr. No.	Receptor Type	Frequency N=130	Percentage (%)
1	ER+/PgR+	40	30.8%
2	ER-/PgR-	63	48.5%
3	ER+/PgR-	19	14.6%
4	ER-/PgR+	8	6.1%
Total		130	100%

## Discussion

Breast carcinoma is the most common malignancy in females and the second leading causing of death due to cancer worldwide [1]. All women are at risk of developing breast cancer regardless of their racial or ethnic origin or heritage [4]. In Pakistan, most of the cases present at a young age and advanced stage, contrary to the western world where it is commonly seen only after 60 years of age [9]. Pakistani women have the highest incidence rate of breast cancer in Asia accounting for one case in every nine women [3-4,9]. 

The management and prognosis of breast cancer is dependent upon many clinicopathologic variables. The histological type, grade, and stage, tumor necrosis, lymphovascular invasion, skin and nipple invasion, lymph node involvement, status of ER, PgR and HER2, BRCA 1 status, cell proliferation marker (ki-67), type of therapy, local recurrence, and gene expression proliferation are all well-known prognostic and predictive markers [4-5,8]. Considering the heterogeneity of breast carcinoma, many prognostic and predictive markers have been purposed to determine tumor behavior. A predictive marker provides information about a patient’s response to treatment while a prognostic marker indicates the overall survival of the patient regardless of the therapy. Expression of ER and/or PgR is a very important prognostic factor and indicates a better outcome than tumors with negative status for ER and PgR [1,9-10]. ER and PgR positive tumors are generally better differentiated and present at an early stage. Adjuvant hormonal therapy is advocated for all the women with positive ER/PgR status regardless of their age, menopausal status, grade, stage, and axillary lymph nodes status or tumor size [7-8,10]. Positivity for ER and PgR receptors in invasive breast carcinoma is found to be 70%-80% and 60%-70%, respectively [7-8]. In Asian countries, the prevalence of hormone receptor-positive tumors is lower than that in the western world [2-3]. However, the number of studies performed on the Asian population is also much less as compared to the west.

In our study, a total of 130 patients of modified radical mastectomy were included, out of which 98.4% were females. The age at the time of diagnosis ranged from 24 to 78 years, the majority (53.1%) of the cases being less than 50 years of age. This is comparable to the study performed in Pakistan and India, where 56.8%, 47.6%, and 58.5%cases were ≤50 years of age, respectively (Table 2). Zeeshan et al. found that 27.4% of breast cancer patients were even younger than 40years [7,11-13].

Invasive ductal carcinoma, not otherwise specified (IDC-NOS), constituted the most predominant histological type, accounting for 124 (95.4%) cases (Figure 1). Similar results were documented in earlier reports, with IDC-NOS being the most common histologic type of breast cancer [2,7,10-11].

According to western studies, ER expression has been found in 50%-80% of cases, and PgR expression is found in 60%-70% of cases of invasive ductal carcinoma [14]. In our study, ER and PgR positivity was found in 59 (45.4%) and 48 (36.9%) cases, respectively. The level of positive expression of hormone receptors was found to be low as compared to a previous study on the Pakistani population by Mahmood et al., which declared 64.1% positivity for ER and 60.6% positivity for PgR [12]. Similarly, a study in Ahmedabad, India, showed a 56.9% positive ER status and a 35.5% PgR positive status [10]. Singh et al. reported ER positivity in 44.6% cases and PgR positivity in 40.4% cases [2]. Hathila et al. reported 53.3% of breast cancer patients positive for ER and 36.6% of patients positive for PgR [15]. The low level of expression of hormonal receptors may be related to a higher grade and/or advanced stage at disease presentation. However, to validate this statement, larger studies are required.

According to the results of hormone receptors, all the cases were divided into four groups: (ER+, PgR+), (ER+, PgR-), (ER-, PgR+), and (ER-, PgR-). In this study, positivity for both the receptors was seen in 40 (30.8%) of cases, while both receptors were negative in 63(48.5%) cases. The ER+, PgR- and ER-, and PgR+ groups had 19 (14.6%) and 8 (6.1%) cases, respectively (Table 3, Figure 2). In our set-up, the majority of breast cancers were negative for ER and PgR, especially in young females, which is contrary to western studies but comparable to studies done in the Asian population [2-3,5]. This is because most of the cases present at a very advanced stage of breast cancer. The advanced stage of breast cancer presentation in Pakistan is due to a lack of early screening programs, lack of awareness about screening programs, and low socioeconomic status [3]. Nabi et al. showed that 59 (42.4%) cases were ER+PgR+, 60 (43.1%) cases were ER-PgR-, 10 (7.1%) cases were ER-PgR+ and ER+PgR-, respectively [7]. Hathila et al. reported co-expression on of ER and PgR in 53.3% cases [15].

In our study, Grade III predominated with 66 (50.77%) cases while previous studies in neighboring regions showed Grade II as the most predominant grade [1,6,7,10-11]. High-grade tumors were mostly negative for hormonal receptors, a finding consistent with that reported by Hashmi et al. [6]. The majority (56.92%) of the tumors belonged to the T3 category of tumor size having size >5cm The 33.85% of the tumors belong to the T2 category, with tumor size dimension between 2 cm and 5 cm. This result is different from the other studies where most of the cases belong to the T2 category of tumor size [1,6-7,10]. The difference may be due to late presentation in hospital, illiteracy, lack of resources, and financial problems.

Lymphovascular invasion and tumor necrosis are important parameters to determine tumor recurrence after treatment [6]. In our study, the lymphovascular invasion was observed in 53 (40.8%) cases. The majority of these tumors belonged to ER- and PgR-. This is in accordance with the results of the study done by Hashmi et al [6]. Similar results were reported by Nabi et al.; the lymphovascular invasion was seen in 35.2% of cases, with the majority belonging to the ER-/PgR- subgroup [7]. A study done in India showed lymphovascular invasion in 55.90% cases, with ER-/PgR- predominance [16]. Tumor necrosis was present in 58 (44.6%) cases. This histological feature was also mostly seen in the ER- and PgR- hormone status. Shrigondekar et al. reported tumor necrosis in 47.85% of tumors in their study [16].

One of the indicators of systemic adjuvant therapy is lymph node involvement by the tumor. It also determines the survival of the patients [3,12]. In this study, 90 (69.2%) cases showed metastatic tumor deposits in axillary lymph nodes. Axillary lymph node metastasis had been recorded in 66.9%, 48.64%, and 61.3% cases in studies done by Nabi et al., Shrigondekaret et al., and Dayal et al., respectively [7,10,15]. Ten cases (7.7%) showed the involvement of more than nine lymph nodes, and these were ER- and PgR- in nine (6.9%) cases. A previous study in Pakistan has also shown less prevalence of lymph node metastasis in hormone receptor-positive tumors [3,6].

Our study showed that most of the breast cancer patients were young and showed lower hormonal positivity than that reported in the western population. The association of the expression of hormone receptors with other prognostic factors was demonstrated. ER- and PgR- tumors showed a higher histologic grade, greater tumor size, and more lymph node involvement by metastasis.

Limitations of the study

1. The patients presenting in our set-up mostly belong to a poor socioeconomic status and were non-affording for immunohistochemistry for ER/PgR and HER2.

2. Most of the patients present in the advanced stage of cancer primarily due to lack of awareness, insufficient screening programs, and inadequate diagnostic facilities. Delayed diagnosis and advanced stage might itself have implications on receptor positivity.

## Conclusions

Low hormone receptor positivity is associated with young patients, advanced stage at presentation, and higher grade in our population. The tumor characteristics are different as compared to the western population. This suggests more consideration into the screening, early diagnosis, and molecular or immunohistochemical typing of this cancer in our population. Moreover, larger studies are required in our population to validate the correlation of clinicopathologic parameters.
